# Design of Culturally and Linguistically Tailored Nutrition Education Materials to Promote Healthy Eating Habits among Pakistani Women Participating in the PakCat Program in Catalonia

**DOI:** 10.3390/nu14245239

**Published:** 2022-12-08

**Authors:** Saba Mohamed-Bibi, Cristina Vaqué-Crusellas, Núria Alonso-Pedrol

**Affiliations:** 1 Department of Social Anthropology, Faculty of Geography and History, University of Barcelona, 08001 Barcelona, Spain; 2 Research Group M3O, Methodology, Methods, Models and Outcomes of Health and Social Sciences, Faculty of Health Sciences and Welfare, University of Vic-Central University of Catalonia, 08500 Vic, Spain; 3 Department of Endocrinology and Nutrition, Health Sciences Research Institute & University Hospital Germans Trias i Pujol, 08916 Badalona, Spain

**Keywords:** food education program, culturally tailored program, health promotion, educational materials, Pakistani women, immigrant

## Abstract

(1) Background: Pakistani women are among one of Catalonia’s most affected groups by obesity and cardiovascular disease. The design of health education strategies for them has become a compelling need. This paper aims to enlighten the elaboration and evaluation procedure of culturally and linguistically tailored nutrition education materials for Pakistani women participating in the PakCat Program, which aims to evaluate the efficacy of a nutrition education strategy allowing the participants to become ambassadors of healthy eating habits for their community. (2) Methods: In this Randomised Control Trial (RCT), 137 Pakistani women (70 from the intervention and 67 from the control group) took part. We conducted 10 sessions for the intervention group and 3 sessions for the control group in the form of small groups. The sessions were conducted in Urdu and Punjabi, and the material was translated into Urdu, Catalan, Spanish and English. For some sessions, we elaborated on new materials and for others, we adapted them from the existing nutrition material in aspects of language and culture. We evaluated the nutritional material from the observation carried out by the dietician who developed the sessions, participants’ feedback at the end of the sessions and a satisfaction questionnaire. (3) Results: We summarised the elaborated material in form of two multilingual nutritional guidelines about portion size and heart-healthy foods. We also registered several materials generated for the PakCat program such as an infographic about myths and beliefs related to food, a booklet to read and interpret the food labels, a recipe book for healthy snacks, and an infographic of 10 tips for healthy eating. We also organised a PhotoVoice exhibition of 70 healthy plates elaborated by the intervention group participants. The participants highly appreciated the material in terms of visualization, cultural and linguistic adequacy, and level of comprehension through all three evaluation methods. (4) Conclusions: The design of culturally and linguistically tailored nutrition education material for Pakistani women living in Catalonia is attainable and effective to meet their specific needs. The healthy dietary recommendation can be adapted to them preserving their traditional dietary pattern, and they acquire the confidence to start following a healthy diet.

## 1. Introduction

The migratory movement of individuals of Pakistani origin toward Spain began in the 1970s, but the most significant growth occurred in 2001 thanks to the flexibility in the processes of regularization of immigrants [1]. Currently, immigrants of Pakistani origin are one of the fastest-growing ethnic groups in Spain, with a current population of about 99.352 [2]. Most of them (56%) reside in Catalonia, especially in the city of Barcelona and its surroundings [3]. The women of this community, who mainly arrive to Catalonia through the family reunification procedures conducted by the male members of the family (husband or father), are still a minority (29%) [1,2,3,4]. The cultural and linguistic barriers hinder them from socializing and utilising different services and community spaces of their cities, and as a result, immigrant women of Pakistani origin are among one of Catalonia’s most invisible ethnic groups [5].

Although the socio-demographic and socio-economic profile of the Pakistani population living in Catalonia is well known [4,5], the Catalan context owns very limited data about the health and nutritional aspects of this ethnic group. Therefore, this population’s growth and the rise of their specific health issues have generated interest in discovering their socio-sanitary profile. As a result, recently, the first retrospective cohort study to evaluate the prevalence and incidence of cardiovascular risk factors and cardiovascular disease (CVD) among Asian immigrants was conducted in Catalonia [6]. This study included 121.000 first-generation Asian immigrants of Indian, Pakistani, Bangladeshi, Filipino, and Chinese origin along with 5.3 million native individuals [6]. It observed that the prevalence of cardiovascular risk factors such as type 2 diabetes (T2D), hypertension, hyperlipidaemia, and obesity along with CVD is much higher in the immigrant population originating from South Asia (SA) (Pakistan, India, and Bangladesh) compared to natives and immigrants from China and Philippines [6].

Although this data is very recent in the Catalan context, some other countries with a larger SA population, such as Norway [7,8], the United Kingdom [9,10], the United States [11,12], and Australia [13], have this information from decades. It is well established that the SA immigrants are affected by CVD at alarmingly younger ages in comparison with the western population as 25% of myocardial infarctions occur in SA under 40 years of age and more than 50% of deaths from CVD occur in SA under 50 years of age [14].

Currently, the prevalence of metabolic syndrome (MetS) which represents the grouping of hyperglycaemia, hypertension, dyslipidemia and central obesity [15] is estimated to be 50% in the United States and 40% in the United Kingdom in SA immigrants [15,16]. However, it is observed that the prevalence of MetS and CVD is higher in SA women as compared to men [6,7,8,9,10,11,12,13,14,15,16,17]. This difference is more significant in immigrants from Pakistan [6,7,8,9,10,11,12,13,14,15,16,17]. In Catalonia, the prevalence of T2D, hypertension, obesity, and heart failure is higher in Pakistani women as compared to Pakistani men [6]. Furthermore, immigrant women of Pakistani origin are among the most affected groups by obesity and heart failure compared to other SA groups and the indigenous populations of Catalonia [6].

Apart from the genetic and metabolic factors [18,19], dietary and lifestyle changes [7,8,9,10,11,12,13,14,15,16,17,18] coupled with acculturative stress and social isolation [20] are some of the reasons that account for the high prevalence of MetS and CVD in Pakistani women living abroad. Consequently, their physical and mental health deteriorates throughout their stay in western countries [6,7,8,9,10,11,12,13,14,15,16,17,18,19,20,21].

Faced with this situation, different countries have successfully designed and implemented culturally and linguistically appropriate food and lifestyle interventions targeting specifically immigrant women of Pakistani origin [13,14,15,16,17,18,19,20,21,22]. However, these types of programs were non-existent in the Catalan context. Thereupon, we designed the first culturally and linguistically appropriate food education program called PakCat [23] based on the Transtheoretical Model [24] to improve the eating habits of Pakistani women living in Catalonia to empower them to become ambassadors of healthy eating habits in their community [23]. The objectives of this paper are to (a) illustrate the elaboration procedure of culturally and linguistically tailored nutrition education materials that we created for Pakistani women who participated in the PakCat program [23] and to (b) evaluate the adaption and complementation of the existing nutrition material in terms of language and culture.

## 2. Materials and Methods

We developed community-based participatory research (CBPR), through a Randomised Control Trial (RCT) which was evaluated and approved by the University of Barcelona Bioethics Committee (CBUB). The protocol and the details of the methodology have been previously published [23]. A total of 137 Pakistani women residing in the Barcelona area participated in this study, of which 70 were assigned to intervention and 67 to the control group. Food education sessions were conducted in small groups of 12–15 women.

### 2.1. Cultural and Linguistic Adaptation of Nutrition Education Material for the Intervention Group

The intervention group participated in 10 educative sessions during10 weeks. Each weekly session had a duration of 60–90 min. The sessions were carried out in Urdu and Punjabi and the material was elaborated in Urdu, Catalan, Spanish and English languages. Every session was based on a stage and process of change (Table 1).

#### 2.1.1. Session 1: Why Us? The Most Common Health Problems of the Pakistani Population

We started the session by presenting the CVD risk factors and their prevalence in SA immigrants. We showed recent news from a Pakistani newspaper which was reporting that according to the International Diabetes Federation (IDF), Pakistan has become the world’s third country with the highest number of patients suffering from diabetes [25].

To highlight the importance of diet and lifestyle in managing the CVD risk factors, we projected a video from the *Dil Se* video series of Advocate Lutheran Hospital (Park Ridge, NJ, USA) [26] in English in which a SA patient explained that he had a second heart attack at the age of 45 years even though he was taking his medication regularly. He explained that a healthy diet and regular exercise have improved his health. This video also mentioned that SA have a 4 times greater risk of heart disease and are affected by it at very young ages.

Hereafter, we explained that the prevalence of MetS and CVD is higher in immigrant women of Pakistani origin as compared with men. Then, we shed light on the causing factors, especially emphasizing the environmental factors (diet, exercise, and migratory grief).

After that, we showed a short video of Diabetes UK in English, in which a Pakistani patient shared his successful experience of managing T2D by incorporating small changes in his diet and lifestyle [27]. Even though most of the participants were able to understand English, we paused both videos after every few minutes to translate them into Urdu and Punjabi in order to clarify the content.

To end the session, we asked the participants to elaborate a list of dietary and lifestyle changes that they could do to improve their health.

#### 2.1.2. Session 2: Food Myths and Beliefs: What Does Science Say?

**Figure 1:**
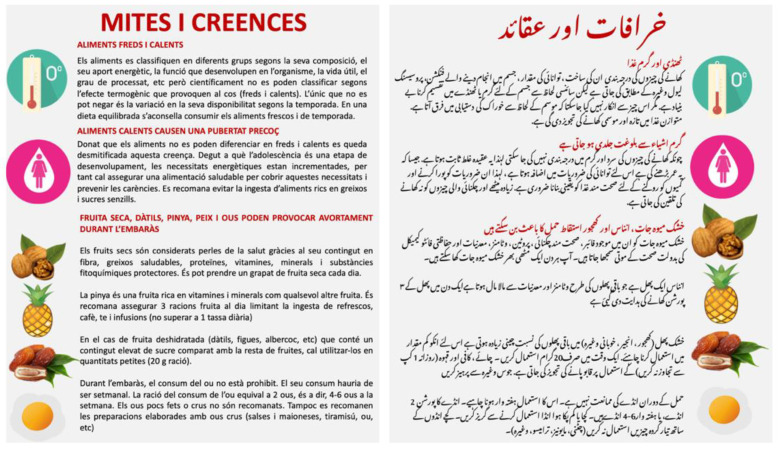
Infographic on myths and beliefs related to food.

In this session, we asked participants’ opinions regarding every myth and belief compiled from the focus groups techniques [23]. Once the discussion was carried out, we presented an infographic (Figure 1) that contained scientific evidence regarding the main myths and beliefs. As most of their myths were related to the life cycle of women, we explained the dietary recommendations for all the stages of a woman’s life (menstruation, pregnancy, breastfeeding, etc).

Throughout the explanation, participants were encouraged to express their concerns and questions. At the end of the session, we handed over the printed infographic to every participant.

#### 2.1.3. Sessions 3: What Is a Healthy Diet?

We started this theoretical-practical session by explaining the definition [28] and benefits of healthy eating [29]. Then, we introduced the concept of nutrients and their functions in our body. We used photographs of traditional Pakistani foods to explain the sources of macro and micronutrients. We also showed videos from the *Dil Se* video series that, apart from explaining the sources of carbohydrates and protein from the SA diet, explained that most SA live a high-carbohydrate and low-protein lifestyle [30,31] that can lead them to poor metabolic control. The video also indicated some healthy sources of protein (lentils and white meat) and suggested increasing their consumption.

**Figure 2:**
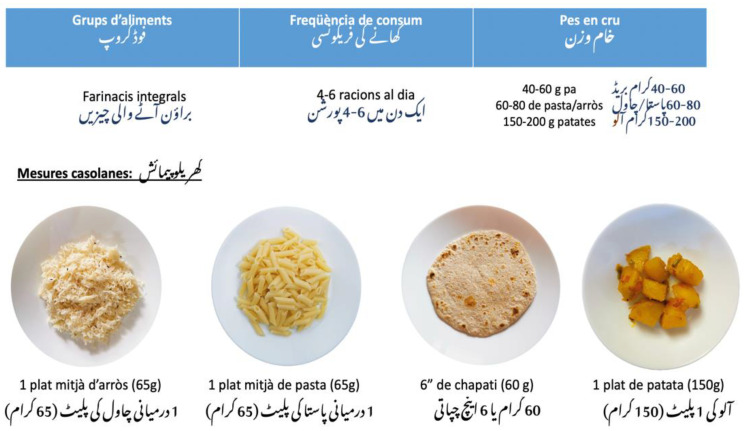
Example of the explanation for serving size and frequency of consumption.

To explain the concept of daily, weekly, and occasional consumption of different food groups, we presented the food pyramid of the Public Health Agency of Catalonia [32]. Following the Catalan [28,29,30,31,32] and Pakistan [33] dietary guidelines, we explained the serving size and the frequency of consumption of different food groups (Figure 2). We presented pictures of the correct portions of different food groups considering participants’ traditional cooking and consumption style.

After the explanation, we did 3 short group activities in which through different techniques (true/false, and multiple-choice questions) we revised the worked material. Then we handed over a visual booklet of serving sizes and frequency of different food groups translated into Urdu.

To end the session, we did a brief joint discussion on the impact of the dietary habits of parents especially mothers on the eating behaviours of their children. We recalled the role of Pakistani women as role models for their families in aspects of health and nutrition.

#### 2.1.4. Sessions 4: The Value of Our Traditional Pakistani Diet

In this session, we recapitulated all the healthy points of the traditional dietary pattern of Pakistan, such as the high consumption of seasonal raw and cooked vegetables, adequate intake of a large variety of pulses (beans, lentils, chickpeas), use of different types of cereals (wheat, millet, corn, etc.), use of an enormous variety of herbs and spices, etc. Then we showed a slide highlighting changes in traditional dietary patterns due to acculturation in Catalonia.

**Figure 3:**
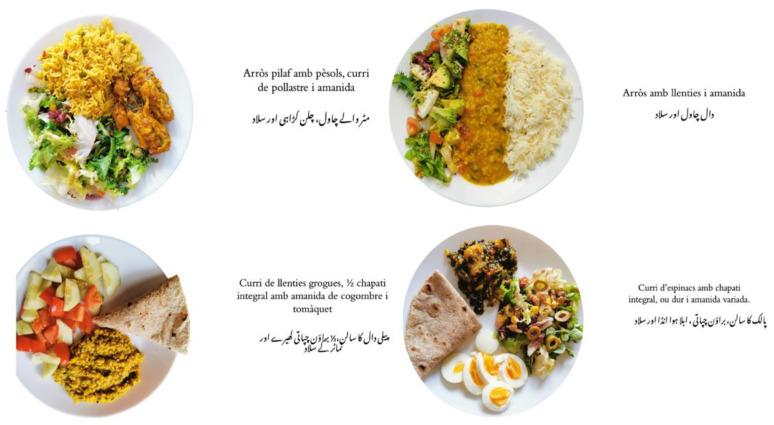
Examples of healthy plates elaborated with traditional Pakistani food.

Hereafter, as a suggested improvement, we presented the structure of Harvard’s Healthy Eating Plate in the Urdu version [34], and we presented 18 images of healthy plates that were elaborated taking into account the traditional and modern culinary techniques, the recommended portion size, the food groups combinations, and the taste preferences of the participants (examples: small chapati of oatmeal and chickpea flour with chicken curry and mixed salad, brown pasta with red beans and mixed vegetables, rice with daal (lentils) and mixed salad, steamed fish and vegetables with yoghurt sauce and small multigrain chapati, etc.) Each plate was discussed in detail empathising especially on its nutritional composition (Figure 3).

We also shared many ideas to complement their plates for different meals. As a final activity, we asked the participants to design a healthy plate and share it with their colleagues who had to evaluate their plate regarding the food groups and serving sizes. At the end of the session, we handed over a visual booklet with explanations and images of 18 healthy plates.

#### 2.1.5. Sessions 5: Small Changes to Eat Better (MORE)

**Figure 4:**
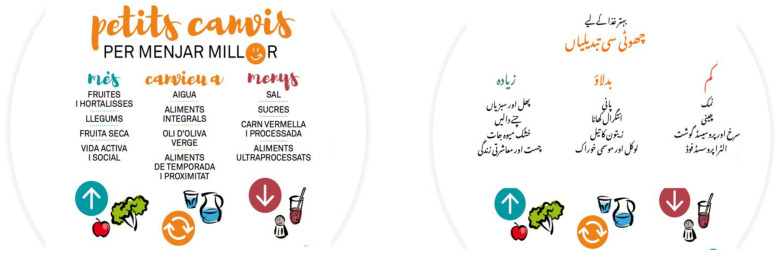
Translation of “*Petits canvis per menjar millor* (small changes to eat better)”.

From this session, we started the presentation of a Catalan guideline called “*Petits canvis per menjar millor* (small changes to eat better)”. This guide remarks on three main aspects: the foods we should increase, the foods that we should change, and the foods we should reduce (more, change, and less) in order to follow a healthy diet (Figure 4). We centred the session on the type of foods we may increase in our diet. We adopted the recommendations of this guide to our participants in terms of language and culture.

We started the session by explaining the benefits of eating fruit and vegetables. After revising their portion and frequency of consumption, we suggested different ideas to increase their consumption. We also showed some video recipes of vegetables from Pakistan and Spanish cooking channels [35,36]. Then, we discussed the season of different fruit and vegetables by presenting their calendar [37]. We clarified that the season may vary from one country to another.

Then we talked about the benefits of consuming legumes and dry fruit. We revised their portion and frequency of consumption, and we shared different ideas to increase their intake. For the next session, we asked participants to prepare and bring some healthy snacks prepared with fruit, vegetables, legumes, and dry fruit.

To end the session, we discussed the importance of staying socially and physically active. We suggested simple and effective ideas to increase physical activity such as short active pauses. For elderly women or those with reduced mobility, we suggested some exercises that can be carried out sitting on a chair [38].

#### 2.1.6. Small Changes to Eat Better (CHANGE)

This session focused on changes we should make in order to follow a healthy diet. We suggested replacing juices and carbonated beverages with water. We explained drinking water’s benefits and proposed ideas to increase water intake. We also presented the urine colour chart to check the hydration level [39].

Hereafter, we recommended substituting the refined wheat flour with whole-wheat or other types of flours such as chickpea, mill, and oat flour to make chapati. We suggested the use of brown or lentil pasta instead of white, and we recommended moderating the consumption of white rice. We showed pictures of all the mentioned products indicating different grocery stores from where they can purchase them.

We talked about the different types of fats, and we suggested using extra virgin olive oil for cooking and seasoning. Finally, we explained the importance of consuming local and seasonal food, and we indicated the labels to identify them. We also revised the seasonal calendar of fruits and vegetables. We indicated some nearby markets from where they can buy fresh, local, and seasonal food. At the end of the session, participants presented their healthy snacks elaborated with fruit, vegetables, legumes, and dry fruit as a substitute for unhealthy food.

#### 2.1.7. Small Changes to Eat Better (LESS)

**Figure 5:**
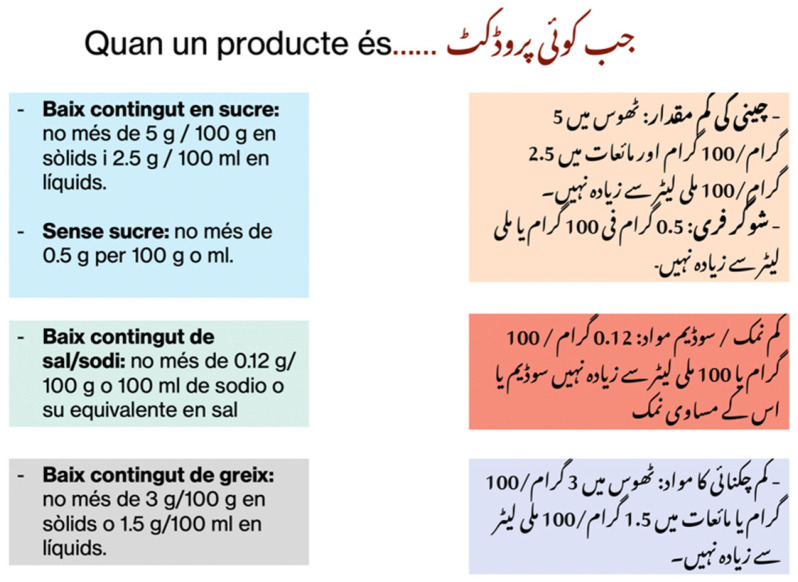
Explanation to analyse and interpret food labels.

This session allowed to work on the foods that should be reduced to follow a healthy diet. We exposed the harmful effects of the excessive use of salt, sugar, red and processed meat, and ultra-processed food. We presented healthy alternatives to reduce their consumption. NOVA classification was used to explain the processing level of different food items [40]. Then we taught about the interpretation of nutrition labels [41]. We also mentioned the quantity from which a product is considered low or high in sugar, salt, and fat (Figure 5).

In this session, we also introduced the PhotoVoice activity that would be worked on in our last session.

#### 2.1.8. Let us Plan Our Weekly Food Purchase!

**Figure 6:**
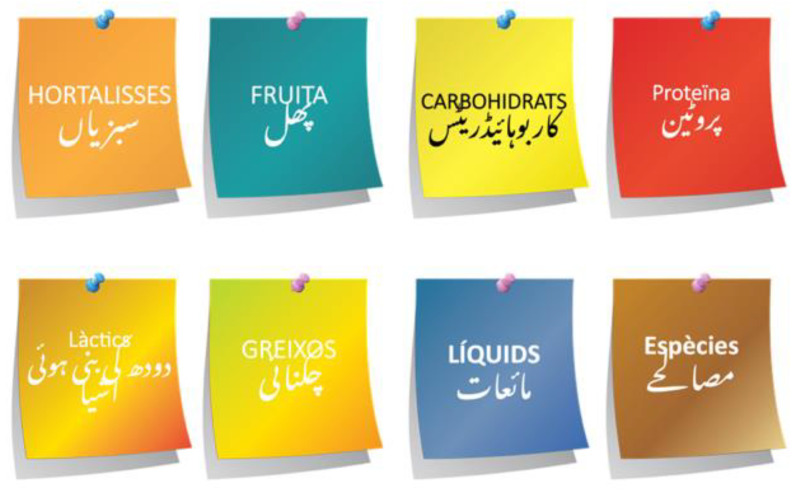
Checklist of food groups for daily/weekly food purchasing.

To start the session, we shared some general recommendations to take into account before going to buy the food such as planning a daily or weekly menu, revising the fridge and pantry to prepare a list of required food items and avoiding going with an empty stomach. Then, we revised the frequency of consumption of each food group. Hereafter, we analysed different products of every food group available in the grocery stores (Figure 6). For the analysis, we showed pictures of the food product and discussed their ingredients, labels, and nutritional value (this information was obtained from the web pages of different local markets). At the end of the session, we prepared a weekly shopping list conjointly, and we handed over a booklet in Catalan and Urdu with explanations and examples to analyse and interpret the food labels.

#### 2.1.9. How to Plan a Balanced Menu?

In this session, to reinforce the knowledge acquired during the 7th and 8th sessions, we elaborated a healthy menu. Following the local nutrition guidelines [28,29,30,31,32], we suggested having 4–5 meals every day (3 main meals and 2 snacks). For breakfast and mid-morning snacks, we suggested the presence of one item from the following food groups: dairy, starch, protein, seasonal fruit, and nuts.

We shared pictures of different ideas for breakfast combining Pakistani and Spanish cuisine such as crêpe of chickpea flour with vegetables and tea with milk without sugar or sweeteners, chapati of oat flour with an omelette, etc. For lunch and dinner, we suggested elaborating a healthy plate following the structure of Harvard’s Healthy Eating Plate [34] that we previously worked on in our fourth session. We also suggested incorporating healthy snacks elaborated with fruit, vegetables, legumes, and dry fruit between lunch and dinner. We remarked on the importance of water as the main beverage in the diet.

**Figure 7:**
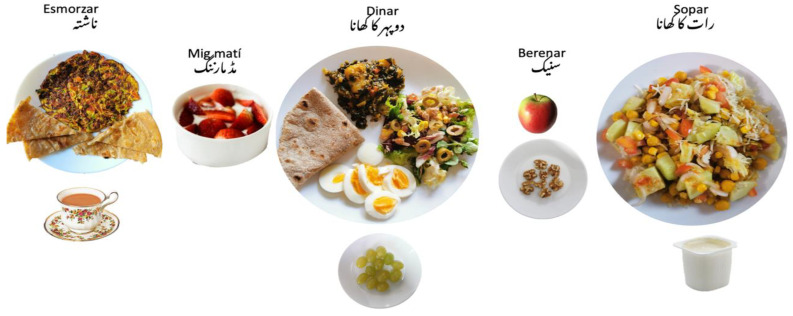
Example of a qualitative one-day menu.

We asked the participants to fill out a 24 h diet recall to use it as a base to prepare the daily healthy menus and to evaluate their nutritional adequacy. We reviewed all the menus conjointly. To reinforce the importance of the food portion, we created an adapted portion size guide specifically for the Pakistani community [42]. After that, summarizing all the material, we presented seven visual examples of daily healthy menus (Figure 7), which later were handed over to participants in the format of a printed booklet. We personalized the dietary recommendations for some participants with different pathologies.

#### 2.1.10. Photovoice

As the last session of the PakCat program, we invited the participants to send a picture of a cooked healthy plate one week before this session. They had to prepare the plates with their favourite food items and a reflection linked to experiences, sensations, emotions, thoughts, or some idea related to the plate in any language. Therefore, we created a PowerPoint with all the material. Every slide contained the name of the participants, the picture of the plate, and the words associated with it.

Participants, one by one, presented their plates explaining the ingredients, portion size, the way of preparation, and the words related to them. They had the opportunity to share this in front of their colleagues and other hospitals and municipal council guests because we invited them to the last sessions of every group.

### 2.2. Cultural and Linguistic Adaptation of Nutrition Education Material for the Control Group

**Figure 8:**
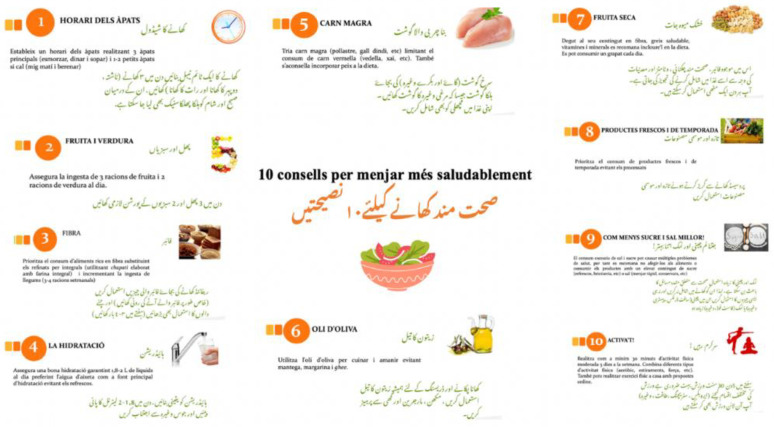
Infographic of 10 tips for healthy eating.

We did 3 basic education sessions with the control group. For the first session, we used the same introductory material that was elaborated for intervention groups. For the second one, we gave 10 general nutritional tips for a healthy lifestyle such as establishing a meal schedule, eating 5 pieces of fruit and vegetable every day, drinking more water, etc. At the end of the session, we handed over an infographic with the 10 tips in Urdu and Catalan (Figure 8). For the last session, we repeated the 8th session of the intervention group “Let’s plan our weekly food purchase!”.

## 3. Results

PakCat program generated several culturally and linguistically appropriate nutrition education materials. Although before starting the program a pilot test was carried out to evaluate the newly generated or adapted materials, during the implementation of the educational sessions some improvement proposals along with the most highly rated elements of the program were identified. The evaluation procedure of the material consisted of three parts: (a) the observations made by the dietician who conducted the sessions and took notes on participants’ reactions and concerns during the sessions; (b) a discussion with the participants about the worked material before the end of each session; (c) a satisfaction questionnaire at the end of the program to evaluate the visualization, cultural adequacy, linguistic adequacy, and comprehensive level of the material. Table 2 summarises the evaluation carried out by the dietician and the feedback of participants regarding worked material in every session.

In the satisfaction questionnaire conducted at the end of the program, participants from the intervention and control group rated excellently all the aspects of the material (visual, culturally adapted, linguistically adapted, and comprehensive level).

To collect a huge part of the nutritional material that we created for the PakCat programme, we designed two guides: (A) A practical guide on food portions for the Pakistani population, and (B) “*Guia d’alimentació cardiosaludable per a la població pakistanesa*” (Heart-healthy food guide for Pakistani community).

(A) A Practical Guide on Food Portions for the Pakistani Population.

This guide is elaborated to facilitate the food intake record to the individuals of Pakistani origin as it visualises three different serving sizes of 12 food groups (farinaceous, vegetables, fruit, dairy products, meat, fish, eggs, nuts, oils and fats, sweets and desserts, snacks and fast food, and drinks) considering their dietary patterns and culinary techniques. The quantity of food in the grammage is also mentioned alongside each photograph. The guide also specifies the size of used utensils in centimetres. It also has a glossary section, which defines the culinary terms of the Pakistani diet. The guide is elaborated in 4 languages (Urdu, Catalan, Spanish, and English).

(B) “*Guia d’alimentació cardiosaludable per a la població pakistanesa*” (Heart-healthy food guide for Pakistani community).

This guide is elaborated in Urdu and Catalan languages, and it consists of four parts:

(1) Small changes to eat better.

After introducing the concept of a healthy diet, we summarized three parts of the guide *petits canvis per menjar millor* (small changes to eat better—more, change, and less). We explained the benefits of increasing the consumption of fruit, vegetable, and legumes mentioning their recommended intake. We also included the part of physical and social life indicating the minimum weekly time of moderate and intense physical activity. For the section of “change”, we suggested incorporating water as the main drink and olive oil as the principal fat of the diet. We also suggested changing refined flour with wholegrain flour. We insisted on consuming fresh and local foods visualising the labels to identify them and adding the seasonal calendar of fruit and vegetables. Finally, we explained the benefits of reducing salt, sugar, red and processed meat, and ultra-processed products. We slightly modified all the sections considering all the doubts that were raised by the participants during different sessions.

(2) Portion and frequency of consumption.

This section started with a picture of the food pyramid of the Public Health Agency of Catalonia [35]. Thereupon, we explained the concept of portion and frequency of consumption [35], and then successively, we explained the frequency of consumption along with the serving size in grams and household measures of every food group.

(3) How to put it into practice?

We began this section by suggesting fractionating the daily food intake into 4–5 meals (3 main meals and 2 snacks). For breakfast and mid-morning snacks, we suggested the presence of dairy, starch, seasonal fruit, and nuts. We also mentioned different food items for all these groups and indicated their serving size in household measurements. Hereafter, we added pictures of different options for breakfast and mid-morning snacks. Then, for lunch and dinner, we suggested elaborating on a healthy plate. We explained the structure of Harvard’s Healthy Eating Plate [37] in detail, and we added different pictures of healthy plates elaborated with Pakistani food and culinary methods. We also presented ideas for preparing healthy evening snacks.

(4) Examples of healthy menu.

This section visualised examples of daily healthy menus with meal pictures. We presented different options to fulfil the daily and weekly recommended intake of different food groups with 4–5 meals.

To visualise and appreciate the participants’ efforts during the PakCat Program, we organised a PhotoVoice exhibition of healthy plates, in which 70 participants of the intervention group presented 70 healthy plates elaborated with their favourite food. The exhibition was visited by the control group, and they were assessed and coached by the intervention group. Many recognised experts from the health field also visited the exhibition and appreciated the work of the participants.

## 4. Discussion

The number of individuals of Pakistani origin is continuously increasing in Catalonia [3]. The population growth of this ethnic/racial group along with their emerging health issues are the factors that highlight the need to discover their health aspects. In response to this need, the prevalence of CVD in immigrants of SA origin was described for the first time in Catalonia [6]. Apart from discovering the high susceptibility for developing CVD and MetS of the SA population, it was revealed that women of Pakistani origin are one of the most affected groups of Catalonia by obesity and heart failure [6]. Due to the cultural and linguistic barrier, they are also one of the most invisible groups in Catalonia [5]. In this context, the design of culturally and linguistically appropriate health strategies is of utter importance.

To address this situation, we designed the first food education program (PakCAT program) in which from the recruitment to the evaluation all the material was culturally and linguistically adapted to immigrant women of Pakistani origin living in Catalonia [26].

In the PakCat program, the sessions were conducted in Urdu and Punjabi, and the educational material was also translated into Urdu. All the dietary recommendations apart from being aligned with the local health and nutrition guidelines were adapted to participants in terms of their cooking and consumption style, as we took into account the usual serving sizes, the commonly used culinary techniques, the preferred combination of flavours and textures, and the food preferences of participants. Conducting the sessions in small groups also facilitated the personalization of the recommendations according to the specific pathologies of some participants.

We also used culturally tailored resources to raise the consciousness of their emerging health issues as we presented credible data obtained from Pakistani newspapers about CVD prevalence among people of Pakistani origin. To make the participants identify with the process of change [30], we also showed videos of individuals of Pakistani origin who successfully made improvements in their health with their lifestyle choices.

To put into practice the acquired knowledge during the session and to empower women, we organised an exhibition of healthy plates, in which 70 women from the intervention group presented 70 healthy plates. They also accompanied and assessed different women of Pakistani origin during their visits to the exhibition complying with their role as promoting agents of healthy eating habits. The nutrition education material was summarised in the form of two plurilingual guides along with an infographic and several booklets that will facilitate the health professionals to assess and guide the Pakistani community living in Catalonia. It can also serve as a model to create similar materials for other ethnic groups.

Although the educational materials were evaluated very positively and greatly appreciated by the participants, we observed that a small fraction of them faced difficulties in understanding the idea of serving sizes and frequency of consumption. We believe that a practical workshop in which they could measure the portions themselves would have been helpful. To visualise the content covered during the sessions, the printing and editing of the materials in the format of magnets, tablecloths, aprons, etc., would have been helpful.

Another aspect we identified as improvable is the extension of the practical part of the analysis and interpretation of nutritional labelling by visiting different local grocery stores as was carried out in SAHELI [10,11,12,13,14,15,16,17,18,19,20,21,22,23,24,25,26,27,28,29,30,31,32,33,34,35,36,37,38,39,40,41,42] and PODOSA intervention [13]. We consider that this practice would have facilitated the acquisition of concepts related to analysing and interpreting food labels.

Even though similar studies have been implemented successfully in other contexts [8,9,10,11,12,13,14], the information about the elaboration and evaluation of nutrition education material for immigrants of Pakistani origin is very limited. We believe that the assessment and publication of educational materials can make them reusable and sustainable. Knowing about their elaboration and evaluation procedure can also facilitate the designing of new or complementary nutrition education materials.

The evaluation of the materials effectuated by the participants allows us to think that it is possible to adapt dietary recommendations to different ethnic groups while maintaining their traditional eating patterns. The appropriation of nutrition education material can successfully convey nutrition knowledge and skills to different racial groups, resulting in an engagement strategy to improve adherence to nutrition education programs. The adaption and complementation can also enrich the existing nutrition education material.

We consider that future research is required to assess the impact of the created material on cardiovascular risk factors, anthropometric measurement, and other aspects such as health-related quality of life.

## 5. Conclusions

The design of culturally and linguistically tailored nutrition education material for Pakistani women living in Catalonia is achievable. The dietary recommendation can be adapted to them without modifying their traditional dietary pattern. The adaption of existing nutrition educational material is needed to arouse their interest in health and nutrition strategies. It can facilitate the comprehension of the content and encourage them to express their opinion regarding different aspects of health and nutrition without being judged about their practices. An appropriate material can improve participants’ adherence to a healthy diet and lifestyle yielding improvement in their health.

The publication of educational resources along with their methods of elaboration can facilitate the design of future nutrition interventions for this ethnic group. Future research will evaluate the effectiveness of the designed material by assessing the changes in different variables of the PakCat study. Future trials may assess the impact of the generated material on the metabolic parameters of the immigrants of SA origin.

## Figures and Tables

**Table 1:** Food education sessions based on the Transtheoretical model.

Stage of Change	Objective	Process of Change	Session
**Pre-contemplation**	Raise awareness of the problem by stimulating the possibility of change	Consciousness raising	1. Why us? The most common health problems of the Pakistani population
		Dramatic relief	
		Self-reevaluation	2. Food myths and beliefs: What does science say?
**Contemplation**	Decant the scale towards change	*Consciousness raising*	3. What is a healthy diet?
		*Self-reevaluation*	
		*Self-reevaluation*	4. The value of our traditional diet
		*Environmental reevaluation*	
**Preparation**	Reinforce knowledge to facilitate the change	*Consciousness raising*	5. Small changes to eat better (MORE)
		*Counterconditioning*	
		*Self-liberation*	6. Small changes to eat better (CHANGE)
		*Counterconditioning*	
		*Self-liberation*	7. Small changes to eat better (LESS)
		*Counterconditioning*	
**Action**	Effectuate the change by promoting self-efficiency	*Counterconditioning*	8. Let’s plan our weekly food purchase!
		Stimulus or environmental control	
		*Counterconditioning*	9. How to plan a balanced menu?
		Stimulus or environmental control	
**Maintenance**	Maintain the change	Helping relationships	10. Photovoice
		Social liberation	Acquisition of the role of promoting agent of healthy eating habits for the rest of the community.

**Table 2:** Evaluation of the educational material.

Sessions	Final Material	Dietician’s Observations	Participants’ Feedback
**1. Why us? The most common health problems of the Pakistani population**	A brief habit change sheet in Urdu and Catalan where participants could write down the lifestyle changes that they want to achieve. **Type of material:** Own elaboration.	The PowerPoint presentation helped raise awareness among the participants. They were surprised by the exposed data and felt identified with the people from the videos. Through the habit change sheet, they were able to reflect and record the healthy changes they wanted to incorporate into their lifestyle.	Participants were satisfied with the material of the session. They appreciated the Urdu translation of it. The videos of SA people who improved their health by incorporating small changes in their lifestyle were highly appreciated by the participants.
**2. Food myths and beliefs: What does science say?**	An infographic in Urdu and Catalan that contained an explanation based on scientific evidence regarding all the discussed myths and beliefs. **Type of material:** Own elaboration.	The infographic allowed to discuss and demystify the myths and beliefs in an orderly way. The part of the infographic that highlighted the dietary aspects for the different stages of women’s lives particularly captured their interest.	Participants liked the infographic about myths and beliefs finding it visual, complete, and easy to understand. They found the content of the infographic very appropriate, which demonstrated the scientific explanation of their beliefs without disparaging or judging them.
**3. What is a healthy diet?**	A visual booklet of serving size and frequency of different food groups translated into Urdu and Catalan. **Type of material:** Adapted from the existing material	The PowerPoint permitted to explain the concept of macro and micronutrients in a simple and efficient way. The designed activities facilitated the interaction between participants and allowed us to evaluate the acquisition of the content.	Participants appreciated the use of Pakistani food for the explanation of the concept of portion size and frequency of consumption. They found the designed activities very simple and effective to revise the content worked during the session. They considered the booklet very visual and convenient for everyday use.
**4. The value of our traditional Pakistani diet**	A visual booklet in Urdu and Catalan with the explanation of the healthy plate’s structure along with 18 examples of different healthy plates elaborated with Pakistani food. **Type of material:** Adapted from the existed material	The Urdu translation of Harvard’s Healthy Eating Plate and its application to the Pakistani diet allowed the participants to perfectly understand its structure. Through the projected material participants were able to recall the positive aspects of the traditional Pakistani diet.	Participants easily comprehended the structure of the healthy plate, finding it simple, practical and suitable for them. They appreciated the presented examples of healthy plates finding them delicious, quick to prepare and adapted to their diet. They greatly appreciated the booklet of 18 healthy plates.
**5. Small changes to eat better (MORE)**	A short recipe book in Urdu and Catalan with 18 simple and easy recipes of healthy snacks. The recipes are based on fruit, vegetables, legumes, and nuts. **Type of material:** Own elaboration.	The translation and cultural adoption allowed the participants to understand the recommendations of a local nutrition guide. The video recipes helped them to understand how they can put the proposed suggestions into practice. Through the guide recommendations and some video demonstrations, they were able to know about a large variety of exercises considering their age, strength and mobility.	Participants found the PowerPoint very visual, simple, and easy to understand. After understanding the guides’ recommendations, they agreed with them. The video recipes from Spanish and Pakistan food channels were highly appreciated. They found them easy to prepare and adapted to their taste.
**6. Small changes to eat better (CHANGE)**		The PowerPoint presentation in Urdu and Catalan allowed proposing healthy changes in a simple and organised way. However, some participants seemed a little confused to locate the suggested grocery stores where they could buy different types of flour and fresh and local products. Through the activity of elaborating healthy snacks, participants learnt to make different Pakistan, Middle East, and Mediterranean snacks more healthily.	With the adapted PowerPoint presentation, participants were able to understand all the suggested changes in the nutrition guide. They found the healthy snacks activity very useful and entertaining which allowed them to put into practice the acquired knowledge during the previous session. They appreciated the recipe book finding it visual and practical.
**7. Small changes to eat better (LESS)**	A booklet in Catalan and Urdu with explanations and examples to analyse and interpret the food labels. The booklet also contains instructions to elaborate a list of daily or weekly food purchases. **Type of material:** Adapted from the existing material	The PowerPoint presentation of the session allowed us to discover and discuss all the doubts of the participants related to the consumption of red and processed meat. It also enabled us to explain the theoretical part related to the analysis and interpretation of food labels.	Participants were satisfied with the material of the session. They easily learnt the basic concepts to read and interpret the nutrition labels of the products and they were eager to analyse their favourite products.
**8. Let’s plan our weekly food purchase!**		The theoretical-practical format of the session permitted the participants to put into practice the acquired concepts during the previous session. The booklet allowed them to revise the theoretical part of the analysis and interpretation of food labels. They also learnt how to access the web pages of local markets and analyse and buy different products.	Participants enjoyed the different activities of the session which allowed them to know about the purchasing of healthy options for all the food groups. Some women with a limited educational background found this session a little difficult to understand.
**9. How to plan a balanced menu?**	A booklet with 7 visual examples of daily healthy menus elaborated with healthy snacks (session 5–6) and healthy plates (session 4). **Type of material:** Own elaboration.	The adaption of the menus to the dietary pattern of participants facilitated the comprehension of the content. The visual examples of the daily menus were greatly appreciated by them. The material permitted them to learn to elaborate the personalised menu for themselves.	Participants appraised the worked material finding it very visual and easily comprehensible. They found the examples of menus realistic, practical, and easy to follow. They were grateful to have a printed booklet of visual examples of menus.
**10. PhotoVoice**	A PowerPoint with 70 healthy plates elaborated by the participants. **Type of material:** Own elaboration.	The participants presented their plates confidently in different languages. Some of them were moved to tears while expressing the emotional significance related to the ingredients of their plates. The guests highly appreciated their efforts.	Participants appreciated each other’s work.

## Data Availability

The following are available online at: https://sabasaba381.wixsite.com/pakcat-program/en/educational-material (accessed on 4 December 2022), (1) Practical guide on food portions for the Pakistani population and (2) Heart-healthy diet guide for Pakistani people.

## References

[1] Beltrán-Antolín J., Sáiz-López A. (2007). La comunidad pakistaní en España. *Anuario Asia-Pacífico*; *Barcelona Centre for International Affaris (CIDOB)*. https://www.cidob.org/articulos/anuario_asia_pacifico/2007/la_comunidad_pakistani_en_espana.

[2] Instituto Nacional de Estadística (INE) (2021). Foreign Population by Country of Nationality, Age (Five-Year Groups), and Sex. https://www.ine.es/jaxiT3/Tabla.htm?t=36825&L=1.

[3] Statistical Institute of Catalonia (2021). Foreign Population by Provinces. https://www.idescat.cat/poblacioestrangera/?geo=cat&nac=d426&b=2&lang=en.

[4] Statical Institute of Catalonia (2021). Foreign Population by Age and Sex. https://www.idescat.cat/poblacioestrangera/?geo=cat&nac=d426&b=1&lang=en.

[5] Güell B., Martínez R., Naz K., Solé A. (2018). Barcelonines d’origen Pakistanѐs: Empoderament i Participació Contra la Feminització de la Pobresa.

[6] Satish P., Vela E., Bilal U., Cleries M., Kanaya A.M., Kandula N., Virani S.S., Islam N., Valero-Elizondo J., Yahya T. (2022). Burden of cardiovascular risk factors and disease in five Asian groups in Catalonia: A disaggregated, population-based analysis of 121 000 first-generation Asian immigrants. Eur. J. Prev. Cardiol..

[7] Mellin-Olsen T., Wandel M. (2005). Changes in food habits among Pakistani immigrant women in Oslo, Norway. Ethn. Health.

[8] Andersen E., Burton N.W., Anderssen S.A. (2012). Physical activity levels six months after a randomised controlled physical activity intervention for Pakistani immigrant men living in Norway. Int. J. Behav. Nutr. Phys. Act..

[9] Bhopal R.S., Douglas A., Wallia S., Forbes J.F., Lean M.E., Gill J.M., McKnight J.A., Sattar N., Sheikh A., Wild S.H. (2014). Effect of a lifestyle intervention on weight change in south Asian individuals in the UK at high risk of type 2 diabetes: A family-cluster randomised controlled trial. Lancet Diabetes Endocrinol..

[10] Jenum A.K., Brekke I., Mdala I., Muilwijk M., Ramachandran A., Kjøllesdal M., Andersen E., Richardsen K.R., Douglas A., Cezard G. (2019). Effects of dietary and physical activity interventions on the risk of type 2 diabetes in South Asians: Meta-analysis of individual participant data from randomised controlled trials. Diabetologia.

[11] Gujral U.P., Kanaya A.M. (2021). Epidemiology of diabetes among South Asians in the United States: Lessons from the MASALA study. Ann. N. Y. Acad. Sci..

[12] Kandula N.R., Bernard V., Dave S., Ehrlich-Jones L., Counard C., Shah N., Kumar S., Rao G., Ackermann R., Spring B. (2020). The South Asian Healthy Lifestyle Intervention (SAHELI) trial: Protocol for a mixed-methods, hybrid effectiveness implementation trial for reducing cardiovascular risk in South Asians in the United States. Contemp. Clin. Trials.

[13] Kousar R., Burns C., Lewandowski P. (2008). A culturally appropriate diet and lifestyle intervention can successfully treat the components of metabolic syndrome in female Pakistani immigrants residing in Melbourne, Australia. Metab. Clin. Exp..

[14] Sharma R., Bhairappa S., Prasad S.R., Manjunath C.N. (2014). Clinical characteristics, angiographic profile and in hospital mortality in acute coronary syndrome patients in South Indian population. Heart India.

[15] Aryal N., Wasti S.P. (2016). The prevalence of metabolic syndrome in South Asia: A systematic review. Int. J. Diabetes Dev. Ctries..

[16] Khan S.A., Jackson R.T. (2016). The prevalence of metabolic syndrome among low-income South Asian Americans. Public Health Nutr..

[17] Jafar T.H., Levey A.S., White F.M., Gul A., Jessani S., Khan A.Q., Jafary F.H., Schmid C.H., Chaturvedi N. (2004). Ethnic differences and determinants of diabetes and central obesity among South Asians of Pakistan. Diabet. Med. A J. Br. Diabet. Assoc..

[18] Sattar N., Gill J.M. (2015). Type 2 diabetes in migrant south Asians: Mechanisms, mitigation, and management. Lancet Diabetes Endocrinol..

[19] Gupta M.D., Gupta P., Mp G., Roy A., Qamar A. (2020). Risk factors for myocardial infarction in very young South Asians. Curr. Opin. Endocrinol. Diabetes Obes..

[20] Gask L., Aseem S., Waquas A., Waheed W. (2011). Isolation, feeling ‘stuck’ and loss of control: Understanding persistence of depression in British Pakistani women. J. Affect. Disord..

[21] Himmelgreen D.A., Pérez-Escamilla R., Martinez D., Bretnall A., Eells B., Peng Y., Bermúdez A. (2004). The longer you stay, the bigger you get: Length of time and language use in the U.S. are associated with obesity in Puerto Rican women. Am. J. Phys. Anthropol..

[22] Johansen K.S., Bjørge B., Hjellset V.T., Holmboe-Ottesen G., Råberg M., Wandel M. (2010). Changes in food habits and motivation for healthy eating among Pakistani women living in Norway: Results from the InnvaDiab-DEPLAN study. Public Health Nutr..

[23] Mohamed-Bibi S., Contreras-Hernández J., Vaqué-Crusellas C. (2022). Pakistani Women: Promoting Agents of Healthy Eating Habits in Catalonia-Protocol of a Culturally and Linguistically Appropriate Randomized Control Trial (RCT) Based on the Transtheoretical Model. Int. J. Environ. Res. Public Health.

[24] Prochaska J.O., Velicer W.F. (1997). The transtheoretical model of health behavior change. Am. J. Health Promot. AJHP.

[25] DAWN News (2021). Pakistan Has World’s Third Highest Number of Diabetics. https://www.dawn.com/news/1650860/pakistan-has-worlds-third-highest-number-of-diabetics.

[26] ABC7 New York (2020). Park Ridge Hospital’s Video Series Dispels Myths to Reduce South Asian Community’s Heart Disease Risk. https://abc7chicago.com/south-asian-cardiovascular-center-heart-disease-risk-park-ridge/5874482/.

[27] Diabetes UK (2019). South Asian Living and Diabetes | Community Champions | Diabetes UK. https://www.youtube.com/watch?v=M1qUzgVxzIQ.

[28] Public Health Agency of Catalonia (2018). Petits Canvis per Menjar Millor. https://canalsalut.gencat.cat/ca/vida-saludable/alimentacio/petits-canvis-per-menjar-millor/.

[29] Centers for Disease Control and Prevention. 2021. Benefits of Healthy Eating. https://www.cdc.gov/nutrition/resources-publications/benefits-of-healthy-eating.html.

[30] AdvocateHealthCare (2020). What South Asians should Know about Carbs in Their Diet. https://www.youtube.com/watch?v=94FfDQ-r4Wo&list=PL5R_Kf4cF6TjEl466Ll_w4qOgsmuFzyTP&index=26.

[31] AdvocateHealthCare (2019). Why Should South Asians Eat More Protein?. https://www.youtube.com/watch?v=7UWpjRQSfe0&list=PL5R_Kf4cF6TjEl466Ll_w4qOgsmuFzyTP&index=3.

[32] Public Health Agency of Catalonia (2015). La Priàmide de L’alimentació Saludable. https://salutpublica.gencat.cat/ca/ambits/promocio_salut/alimentacio_saludable/la-piramide-de-lalimentacio-saludable.

[33] Food and Agriculture Organization of the United Nations, Ministry of Panning, Development and Reform, Government of Pakistan (2019). Pakistan Dietary Guidelines for Better Nutrition. https://www.pc.gov.pk/uploads/report/Pakistan_Dietary_Nutrition_2019.pdf.

[34] Harvard TH Chan. School of Public Health (2011). Healthy Eating Plate. https://www.hsph.harvard.edu/nutritionsource/healthy-eating-plate/.

[35] Receta Cubana (2021). Cómo Hacer CREMA DE CALABAZA Casera. https://www.youtube.com/watch?v=S5E2F42BXd8.

[36] Healthy Fusion (2021). Healthy Protein Salad—Weight Loss Friendly by Healthy Food Fusion. https://www.youtube.com/watch?v=_5AMBk5zD-w.

[37] Public Health Agency of Catalonia Aliments Frescos i de Temporada. https://opcions.org/wp-content/uploads/2017/05/calendari_Alimentsfrescos-web.pdf.

[38] Fisioterapia Querétaro Rutina de Entrenamiento en SILLA para ADULTOS MAYORES. https://www.youtube.com/watch?v=67wl05H1K3U&t=631s.

[39] South Tees Hospitals NHS Foundation Trust (2022). Urine Colour Chart. https://www.southtees.nhs.uk/content/uploads/2022/08/Urine-colour-chart.pdf.

[40] Monteiro C.A., Cannon G., Lawrence M., Costa Louzada M.L., Pereira Machado P. (2019). Ultra-Processed Foods, Diet Quality, and Health Using the NOVA Classification System.

[41] Agencia Española de Seguridad Alimentaria y Nutrición (AESAN) (2015). Información Alimentaria Facilitada al Consumidor. https://www.aesan.gob.es/AECOSAN/web/seguridad_alimentaria/detalle/etiquetado_informacion_alimentaria.htm.

[42] Kandula N.R., Dave S., De Chavez P.J., Bharucha H., Patel Y., Seguil P., Kumar S., Baker D.W., Spring B., Siddique J. (2015). Translating a heart disease lifestyle intervention into the community: The South Asian Heart Lifestyle Intervention (SAHELI) study; a randomized control trial. BMC Public Health.

